# Constituents, Pharmacokinetics, and Pharmacology of *Gegen-Qinlian* Decoction

**DOI:** 10.3389/fphar.2021.668418

**Published:** 2021-05-07

**Authors:** Jing-Ze Lu, Dan Ye, Bing-Liang Ma

**Affiliations:** Department of Pharmacology, School of Pharmacy, Shanghai University of Traditional Chinese Medicine, Shanghai, China

**Keywords:** Gegen-Qinlian decoction, traditional Chinese medicine, quality control, material basis, pharmacokinetics, pharmacological effects

## Abstract

*Gegen-Qinlian* decoction (GQD) is a classic traditional Chinese medicine (TCM) formula. It is composed of four TCMs, including *Puerariae Lobatae Radix*, *Scutellariae Radix*, *Coptidis Rhizoma*, and *Glycyrrhizae Radix* et *Rhizoma Praeparata cum Melle*. GQD is traditionally and clinically used to treat both the “external and internal symptoms” of diarrhea with fever. In this review, key words related to GQD were searched in the Web of Science, PubMed, China National Knowledge Infrastructure (CNKI), and other databases. Literature published mainly from 2000 to 2020 was screened and summarized. The main constituents of GQD could be classified into eight groups according to their structures: flavonoid *C*-glycosides, flavonoid *O*-glucuronides, benzylisoquinoline alkaloids, free flavonoids, flavonoid *O*-glycosides, coumarins, triterpenoid saponins, and others. The parent constituents of GQD that enter circulation mainly include puerarin and daidzein from *Puerariae Lobatae Radix*, baicalin and wogonoside from *Scutellariae Radix*, berberine and magnoflorine from *Coptidis Rhizoma*, as well as glycyrrhetinic acid and glycyrrhizic acid from *Glycyrrhizae Radix et Rhizoma Praeparata cum Melle*. GQD is effective against inflammatory intestinal diseases, including diarrhea, ulcerative colitis, and intestinal adverse reactions caused by chemotherapeutic agents. Moreover, GQD has significant effects on metabolic diseases, such as nonalcoholic fatty liver and type 2 diabetes. Furthermore, GQD can be used to treat lung injury. In brief, the main constituents, the pharmacokinetic and pharmacological profiles of GQD were summarized in this review. In addition, several issues of GQD including effective constituents, interactions between the constituents, pharmacokinetics, interaction potential with drugs and pharmacological effects were discussed, and related future researches were prospected in this review.

## Introduction

**FIGURE 1:**
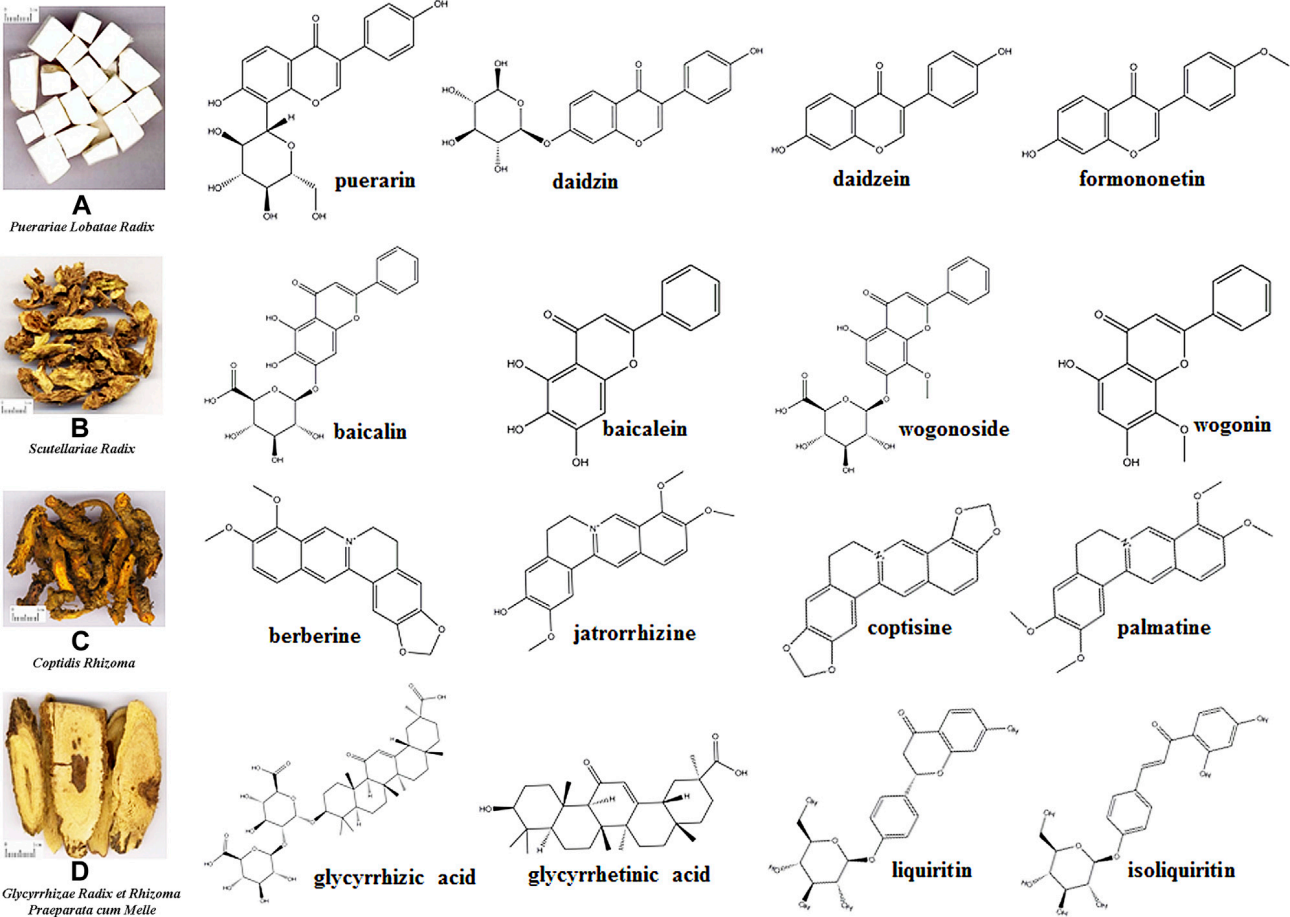
Four traditional Chinese medicines composing *Gegen-Qinlian* decoction and structures of the main constituents.

*Gegen-Qinlian* decoction (GQD) is a classic traditional Chinese medicine (TCM) prescription formulated during the Eastern Han Dynasty rule (25–220). It is composed of four TCMs (Figure 1), including *Puerariae Lobatae Radix* (*Gegen* in Chinese), *Scutellariae Radix* (*Huangqin* in Chinese), *Coptidis Rhizoma* (*Huanglian* in Chinese), and *Glycyrrhizae Radix et Rhizoma Praeparata cum Melle* (*Gancao* in Chinese) in a mass ratio of 8:3:3:2 (Hai and Wei, 2009). GQD is traditionally and clinically used to treat both the “external and internal symptoms” of diarrhea with fever (Hai and Wei, 2009).

Stability and controllable quality are important prerequisites in the modern research and development of TCMs (Song et al., 2013). At present, qualitative and quantitative analyses of the main bioactive constituents of TCMs are few of the main quality control methods for TCMs (Jiang et al., 2010). Owing to the rapid development of analytical methods, including liquid chromatography-mass spectrometry (LC-MS) (Ganzera and Sturm, 2018), more than a hundred constituents have been qualitatively detected (Qiao et al., 2016b; Liu T. et al., 2017), and dozens of constituents have been quantitatively detected in GQD (Wang et al., 2016).

Pharmacokinetics is the study of the absorption, distribution, metabolism, and excretion (ADME) of drugs and their dynamic changes in circulation. The pharmacokinetic study of TCMs faces challenges caused mainly by the huge diversity, low *in vivo* concentration, and unique pharmacokinetic properties of their constituents (Shi et al., 2018). In addition, complex interactions often occur between the constituents of TCMs (Shi et al., 2018). Currently, the pharmacokinetics of dozens of constituents of GQD can be studied simultaneously (Qiao et al., 2018), which helps reveal the effective constituents of GQD and guiding its clinical applications.

Carrying out high-level research on the pharmacological effect and action mechanism of TCMs is an important part in the modernization of TCMs. Some methods that can systematically describe the effects and action mechanism of drugs as a whole have unique advantages in this field. For example, metabolomics investigates the responses of living organisms to pathological stimuli and drug treatments in a holistic manner (Wang M. et al., 2017). This agrees with the holistic thinking of TCMs and has been widely applied in TCM studies (Wang M. et al., 2017). Furthermore, network pharmacology reveals the interactions between compounds and targets by establishing a compound-protein/gene-disease network (Luo et al., 2020). Network pharmacology has recently been used in TCM research, including screening of bioactive constituents, discovery of targets, and prediction of possible action mechanisms (Luo et al., 2020). In addition, after oral administration, the bioactive constituents of TCMs can modulate the structure and metabolism of gut microbiota (Feng et al., 2019). In turn, gut microbiota can transform TCM constituents (Feng et al., 2019). Therefore, gut microbiota analysis is a new Frontier for understanding the action mechanisms of TCMs (Feng et al., 2019). Studies have shown that GQD has antidiarrheal (Liu et al., 2019b; Hua et al., 2019), anti-colitis (Xu B.-L. et al., 2015; Li et al., 2016; Fan et al., 2019; Zhao Y. et al., 2020), anti-nonalcoholic fatty liver disease (NAFLD) (Wang Y.-l. et al., 2015; Guo et al., 2017; Guo et al., 2018; Hao et al., 2020; Zhang et al., 2020), antidiabetic (Zhang et al., 2013; Xu J. et al., 2015; Cao et al., 2020; Tu et al., 2020), anti-lung injury (Ding et al., 2020; Shi et al., 2020), antitumor (Wang N. et al., 2015; Wu Y. et al., 2019; Lv et al., 2019) and other pharmacological effects. Metabolomics (Tian et al., 2013; Hua et al., 2019), network pharmacology (Li et al., 2014b; Cao et al., 2020; Ding et al., 2020; Hao et al., 2020; Xu et al., 2020; Zhong et al., 2020), gut microbiota analysis (Xu J. et al., 2015; Guo et al., 2018; Liu et al., 2019b; Lv et al., 2019), and other methods have been comprehensively used to explain the action mechanism of GQD.

Many breakthroughs have been made in the research of GQD, but no critical review have been published. In this review, key words related to GQD were searched in the Web of Science, PubMed, China National Knowledge Infrastructure (CNKI), and other databases, and important literatures published mainly from 2000 to 2020 were summarized to improve the readers’ understanding of the main constituents, pharmacokinetic properties, and pharmacological effects of GQD.

## Constituents

*Puerariae Lobatae Radix* is the dried root of *Pueraria lobata* (Willd.) Ohwi; it mainly contains isoflavones and isoflavone glycosides, such as puerarin, daidzin, daidzein, and formononetin (Figure 1A) (Wong et al., 2011). *Scutellariae Radix* is the dry root of *Scutellaria baicalensis* Georgi; it mainly contains flavonoids, including baicalin, baicalein, wogonoside, and wogonin (Figure 1B) (Zhao et al., 2016). *Coptidis Rhizoma* is the dried rhizome of *Coptis chinensis* Franch., *Coptis deltoidea* C. Y. Cheng et Hsiao, or *Coptis teeta* Wall; it mainly contains alkaloids, such as berberine, jatrorrhizine, coptisine, and palmatine (Figure 1C) (Wang et al., 2019). *Glycyrrhizae Radix et Rhizoma Praeparata cum Melle* is the dry root and rhizome of *Glycyrrhiza uralensis* Fisch., *Glycyrrhiza inflata* Bat, or *Glycyrrhiza glabra* L.; it mainly contains flavonoid glycosides, triterpenoid saponins, and free phenolic compounds, such as glycyrrhizic acid, glycyrrhetinic acid, liquiritin, isoliquiritin (Figure 1D) (Song et al., 2017). More qualitative and quantitative information of constituents of *Puerariae Lobatae Radix* (Wu W. et al., 2019), *Scutellariae Radix* (Qiao et al., 2016a), *Coptidis Rhizoma* (Ren et al., 2013) and *Glycyrrhizae Radix et Rhizoma Praeparata cum Melle* (Song et al., 2017) can be obtained from the relevant literatures. However, considering the chemical reactions that may exist in the decoction process of TCMs (Wang et al., 2012; Li et al., 2017), the chemical constituents of GQD cannot be regarded as a simple superposition of the constituents of the four individual TCMs. Therefore, systematic qualitative and quantitative studies have been conducted.

### Qualitative Studies

High-resolution mass spectrometry has been widely used in the qualitative study of the complex constituents of TCMs (Wolfender et al., 2015). Comprehensive two-dimensional liquid chromatography coupled with quadrupole time-of-flight mass spectrometry (MHC 2DLC/qTOE-MS) provides a powerful technique for global chemical profiling of TCM formulas (Qiao et al., 2016b). By using the chemical profiling technique, a total of 280 compounds were detected and 125 were characterized in GQD (Qiao et al., 2016b). Among the 125 compounds, 31 compounds were from *Puerariae Lobatae Radix*, 28 compounds were from *Scutellariae Radix*, 45 compounds were from *Coptidis Rhizoma*, and 21 compounds were from *Glycyrrhizae Radix et Rhizoma Praeparata cum Melle* (Qiao et al., 2016b). The multiple heart-cutting mode extends the D-2 modulation time to effectively separate minor compounds (Qiao et al., 2016b). A 12-loop MHC-2DLC/qTOF-MS system allowed the separation and detection of 13 additional minor compounds, which were mainly phenolic compounds (Qiao et al., 2016b). In another study, by using ultra-high performance liquid chromatography (UPLC) coupled with Fourier transform ion cyclotron resonance mass spectrometry (FT-ICR MS), 134 compounds in GQD were identified or tentatively characterized (Liu T. et al., 2017). In brief, chemical constituents in GQD can be classified into eight groups according to their structures: flavonoid *C*-glycosides, flavonoid *O*-glucuronides, benzylisoquinoline alkaloids, free flavonoids, flavonoid *O*-glycosides, coumarins, triterpenoid saponins, and other atypical and abundant backbones (Qiao et al., 2016c).

### Quantitative Researches

Using UPLC-MS/MS, 50 bioactive compounds of GQD, including acidic/alkaline and high-polarity/low-polarity compounds, were simultaneously quantified (Table 1) (Wang et al., 2016). In general, GQD contains abundant glycosides and saponins. In the freeze-dried powder of GQD, the constituents present at a concentration of more than 1 mg/g powder were as follows: puerarin, 6′-o-xylosylpuerarin, 3′-hydroxypuerarin, baicalin, 3′-methoxypuerarin, daidzin, glycyrrhizic acid, wogonoside, genistin, formonetin 8-*C*-glu (6,l)-apioside, genistein 8-*C*-apiofuranosyl (l, 6) glucoside, berberine, chrysin 6-*C*-arabinoside-8-*C*-glucoside, mirificin, and 5,7,6′-trihydroxyflavone 2′-*O*-β-d-glucopyranoside (Wang et al., 2016). Among them, the constituents present at the highest concentration in *Puerariae Lobatae Radix*, *Scutellariae Radix*, *Coptidis Rhizoma*, and *Glycyrrhizae Radix et Rhizoma Praeparata cum Melle* are puerarin, baicalin, berberine, and glycyrrhizic acid, respectively (Wang et al., 2016). The total content of constituents from *Puerariae Lobatae Radix*, *Scutellariae Radix*, *Coptidis Rhizoma*, and *Glycyrrhizae Radix et Rhizoma Praeparata cum Melle* was 60.04, 13.29, 4.27, and 2.47 mg/g powder, respectively (Wang et al., 2016). It should be noted that raw materials (herbal pieces) of different origins and batch sources can significantly affect the content of the constituents of GQD (Wang et al., 2016; Zhong et al., 2016).

**TABLE 1:** Constituents of *Gegen-Qinlian* decoction (GQD) and their pharmacokinetic parameters obtained in rats that received 18.9 g/kg (1.5-fold of the clinical dosage) oral GQD.

Source	Constituents	Contents (mg/g)	T_max_ (h)	C_max_ (ng/ml)	AUC_last_ (h·ng/mL)	T_1/2_ (h)
P	1 puerarin	31.24	1.20 ± 1.49	577.23 ± 56.32	8,611.51 ± 765.80	5.58 ± 2.90
P	2 6″-*O*-xylosylpuerarin	5.89	n.d.	n.d.	n.d.	n.d.
P	3 3′-hydroxypuerarin	5.61	n.d.	n.d.	n.d.	n.d.
P	4 3′-methoxypuerarin	4.77	1.62 ± 0.22	206.44 ± 12.98	5,150.57 ± 441.22	7.59 ± 4.66
P	5 daidzin	4.20	0.88 ± 0.11	27.35 ± 3.34	345.71 ± 30.34	3.96 ± 1.02
P	6 genistin	2.66	1.04 ± 0.78	4.97 ± 1.11	72.56 ± 15.23	6.93 ± 0.73
P	7 formonetin 8-*C*-glu (6,l)-apioside	1.99	n.d.	n.d.	n.d.	n.d.
P	8 genistein 8-*C*-apiofuranosyl (1,6)glucoside	1.32	8.39 ± 0.23	11.27 ± 3.32	98.8 ± 24.76	6.77 ± 3.44
P	9 mirificin	1.14	n.d.	n.d.	n.d.	n.d.
P	10 ononin	0.91	0.86 ± 0.15	34.76 ± 34.18	500.16 ± 233.12	5.73 ± 2.20
P	11 daidzein	0.17	8.00 ± 12.83	186.03 ± 36.23	3,051.89 ± 998.38	6.23 ± 1.36
P	12 3′-methoxymirificin	0.13	0.36 ± 0.03	9.94 ± 3.68	86.26 ± 23.9	1.04 ± 0.04
P	13 formononetin 8-*C*-apiofuranosyl (1,6)glucoside	n.d.	0.76 ± 3.76	16.01 ± 5.44	168.12 ± 83.27	7.03 ± 2.31
P	14 (4S)-puerol B 2′′ -*O*-glucopyranoside	n.d.	0.52 ± 0.26	10.93 ± 9.40	37.47 ± 21.08	7.98 ± 2.88
S	1 baicalin	4.99	7.29 ± 1.38	1,183.39 ± 870.40	22,274.24 ± 11,236.50	4.80 ± 3.27
S	2 chrysin-8-*C*-glucoside	4.20	n.d.	n.d.	n.d.	n.d.
S	3 wogonoside	2.91	7.05 ± 1.27	213.32 ± 39.28	10,342.34 ± 112.34	8.13 ± 2.98
S	4 chrysin 6-*C*-α-L-arabinoside-8-*C*-β- glucoside	1.15	2.34 ± 0.08	11.09 ± 2.84	155.13 ± 13.94	1.62 ± 0.98
S	5 5,7,6′-trihydroxyflavone 2′-*O*-β- glucopyranoside	1.04	n.d.	n.d.	n.d.	n.d.
S	6 oroxylin A 7-*O*-glucuronide	0.78	8.05 ± 1.29	174.96 ± 29.58	6123.69 ± 477.76	8.84 ± 1.40
S	7 chrysin 8-*c*-arabinoside-6-*c*-glucoside	0.39	n.d.	n.d.	n.d.	n.d.
S	8 baicalein 7-*O*-glucuronide	0.33	n.d.	n.d.	n.d.	n.d.
S	9 baicalein	0.27	0.42 ± 0.03	11.11 ± 0.66	108.29 ± 46.96	7.57 ± 0.21
S	10 lateriflorein 7-*O*-glucuronide	0.25	0.44 ± 3.87	48.75 ± 3.88	699.68 ± 77.05	4.33 ± 3.23
S	11 wogonin 5-*O*-glucoside	0.17	2.00 ± 1.29	1.83 ± 0.14	52.99 ± 7.56	16.13 ± 4.22
S	12 wogonin	0.06	7.89 ± 0.99	12.87 ± 7.39	282.08 ± 11.34	6.63 ± 2.38
S	13 norwogonin 7-*O*-glucuronide	0.03	7.05 ± 1.23	379.34 ± 98.54	5,267.25 ± 987.31	4.88 ± 2.87
S	14 chrysin	0.02	n.a.	n.a.	n.a.	n.a.
S	15 5,6′-dihydroxy- 6,7, 8,2′-tetramethoxy flavone	0.02	n.d.	n.d.	n.d.	n.d.
S	16 oroxylin A	0.01	n.a.	n.a.	n.a.	n.a.
S	17 chrysin 6-*C*-β- glucoside-8-*C*-β- arabinoside	n.d.	4.53 ± 0.19	23.2 ± 1.04	229.1 ± 40.33	3.07 ± 1.48
S	18 chrysin 7-*O*-glucuronide	n.d.	6.42 ± 0.34	49.92 ± 6.41	1,023.55 ± 117.36	8.33 ± 6.32
C	1 berberine	1.18	0.39 ± 0.76	28.20 ± 17.45	389.12 ± 187.34	11.42 ± 2.78
C	2 coptisine	0.32	0.74 ± 0.32	6.56 ± 4.03	109.2 ± 73.23	5.20 ± 1.37
C	3 epiberberine	0.29	0.58 ± 0.12	7.21 ± 3.62	29.35 ± 21.32	4.84 ± 0.13
C	4 palmatine	0.29	0.17 ± 0.03	17.15 ± 2.98	68.34 ± 39.23	1.62 ± 1.82
C	5 magnoflorine	0.15	4.13 ± 1.21	19.71 ± 5.67	198.48 ± 74.90	8.35 ± 1.26
C	6 jatrorrhizine	0.12	0.72 ± 0.11	8.54 ± 0.05	194.57 ± 25.67	6.97 ± 1.77
C	7 demethyleneberberine	0.12	n.a.	n.a.	n.a.	n.a.
G	1 glycyrrhizic acid	3.16	6.90 ± 0.28	55.62 ± 14.34	870.76 ± 570.92	4.78 ± 9.98
G	2 22β-acetoxyl-glycyrrhizin	0.53	n.d.	n.d.	n.d.	n.d.
G	3 isoliquiritin	0.18	0.49 ± 1.01	2.59 ± 2.43	11.93 ± 2.4	1.62 ± 0.33
G	4 liquiritin	0.12	2.50 ± 4.76	20.76 ± 1.86	416.94 ± 210.31	12.05 ± 1.16
G	5 liquiritin apioside	0.09	2.09 ± 0.24	12.6 ± 0.14	514.13 ± 7.53	15.43 ± 2.23
G	6 isoliquiritin apioside	0.07	3.68 ± 8.09	3.28 ± 1.23	40.83 ± 10.23	5.35 ± 1.41
G	7 licorice-saponin E2	0.05	n.d.	n.d.	n.d.	n.d.
G	8 licoricidin	0.04	n.d.	n.d.	n.d.	n.d.
G	9 glycyrrhetinic acid	0.02	14.00 ± 3.22	371.14 ± 66.69	8,481.08 ± 1298.09	7.96 ± 2.56
G	10 isoliquiritigenin	0.01	5.33 ± 0.89	2.81 ± 0.87	39.2 ± 8.9	3.55 ± 0.08
G	11 formononetin	0.01	2.92 ± 0.08	8.62 ± 0.39	94.18 ± 32.63	23.78 ± 16.76
G	12 licorice saponin G2	n.d.	3.89 ± 1.41	10.3 ± 6.38	102.23 ± 53.34	2.53 ± 0.14
G	13 glycycoumarin	0	2.00 ± 1.24	7.47 ± 8.1	37.91 ± 29.93	11.35 ± 3.38
G	14 liquiritigenin	0	1.00 ± 0.93	27.35 ± 9.97	153 ± 9.34	11.23 ± 7.69
G	15 glycyrol	0	n.a.	n.a.	n.a.	n.a.
G	16 isoglycyrol	0	n.d.	n.d.	n.d.	n.d.

P, *Puerariae Lobatae Radix*; S, *Scutellariae Radix*; C, *Coptidis Rhizoma*; G, *Glycyrrhizae Radix* et *Rhizoma Praeparata* cum Melle; *n.a.*, the pharmacokinetic parameters are not available due to limited amounts of data points; *n.d.*, related data were not reported.

The data of the content was compiled according to Wang's study (Wang et al., 2016), and the data of pharmacokinetic parameters was compiled according to Qiao's study (Qiao et al., 2018). In order to facilitate readers to quickly understand the main constituents and their pharmacokinetic data, the data were rearranged from high to low according to their contents in GQD.

## Pharmacokinetics

### Absorption

According to the Biopharmaceutics Classification System (BCS), solubility and permeability are important factors affecting the intestinal absorption of drugs (Rinaki et al., 2003). TCMs are usually composed of multiple constituents with different structures. They can affect the properties of a coexisting constituent through physical and chemical interactions. Consequently, the biopharmaceutical properties of some constituents in TCMs may be significantly different from those of the pure constituents. For example, in contrast to pure baicalein, which is a BCS-II substance having low solubility and high permeability, baicalein in GQD has good solubility and permeability and can be considered as a BCS-I substance (Liu Y. et al., 2019). As another example, berberine hydrochloride has low solubility and permeability, belonging to the BCS-IV class; however, berberine hydrochloride in GQD can be classified as a BCS-III compound, that is, a compound with good solubility but poor permeability (Liu Y. et al., 2017).

The results of a transport study in Caco-2 cells showed that only triterpenoid saponins (licorice-saponin G2 and glycyrrhizic acid) had poor permeability (*P*_*AB*_ ≤ 1.50 × 10^–6^ cm/s). In contrast, other constituents, including alkaloids (magnoflorine, demethyleneberberine, coptisine, epiberberine, jatrorrhizine, berberine, palmatine), flavonoid *C*-glycosides [3′-methoxypuerarin, formonetin 8-*C*-glu (6,l)-apioside, genistein 8-*C*-apiofuranosyl (1→6) glucoside, 3′-methoxymirificin, and chrysin-6-*C*-arabinoside-8-*C*-glucoside], *O*-glycosides (daidzin, ononin, wogonin-5-*O*-glycosidase, and liquiritin, isoliquiritin, isoliquiritinapioside), and *O*-glucuronides (norwogonin 7-*O*-gluA, oroxylin A 7-*O*-gluA, wogonoside, baicalin, lateriflorein 7-*O*-gluA, chrysin 7-*O*-gluA), had favorable permeability (*P*_*AB*_ ≥ 1.05 × 10^–5^ cm/s) (Wang Q. et al., 2017).

Metabolic stability also affects the intestinal absorption of oral drugs (Darwich et al., 2010). In particular, gut microbiota is known for its direct and indirect effects on drug and xenobiotic metabolism (Wilson and Nicholson, 2017). The intestinal flora mediates the metabolism of GQD constituents, including daidzin, genistin, and liquiritin *via* beta-glucosidase; baicalin, wogonoside, and glycyrrhizin *via* beta-glucuronidase; and berberine and coptisine *via* nitroreductase (Liu et al., 2019a).

Regarding the sites of absorption, the results of an *in vitro* absorption experiment using everted gut sacs showed that the main constituents of GQD, such as puerarin, daidzein, liquiritin, scutellarin, baicalin, wogonin, berberine, and palmatine, can be absorbed in all segments of rat intestine, with jejunum and ileum as the main absorption sites (Chen et al., 2020).

It is worth noting that in enteritis model rats, the absorption of each constituent was significantly improved (Chen et al., 2020), which is beneficial for increasing its exposure level in the body. For example, the absorption rate constant (K_a_) of puerarin in duodenum was increased from 0.284 ± 0.001 to 0.593 ± 0.003 μg/min/cm^2^. The absorption of each constituent was also significantly improved (*p* < 0.05) in mini pigs with diarrhea induced by *Escherichia coli* (Ling et al., 2018). The decreased activity of drug-metabolizing enzymes (Ling et al., 2018) and efflux transporters (Chen et al., 2020) explained the increased absorption of GQD constituents under pathological conditions.

### Metabolism

After oral administration of GQD, 67 biotransformed constituents were detected in the plasma, urine, bile, and feces of rats (Liu et al., 2018). The metabolites were mainly formed through sulfate and glucuronide conjugation (Liu et al., 2018). In another study, by using a compound-to-extract-to-formulation strategy, 131 GQD metabolites were identified in the biofluids of rats following oral administration of GQD (Qiao et al., 2016c). The metabolic reactions involved included methylation, demethylation, oxidation (hydroxylation), oxidation (dehydrogenation), reduction (hydrogenation), ring cleavage, hydrolysis, glucuronidation, and sulfation (Qiao et al., 2016c; Liu et al., 2018). Sulfate conjugation and glucuronide conjugation were the major metabolic reactions. Specifically, methylation, methoxylation, hydrolysis, hydroxylation, glucuronidation, and sulfation are the main metabolic processes of flavonoids, whereas hydroxylation, hydrogenation, demethylation, glucuronidation, and sulfation reactions are the major metabolic pathways of alkaloids (Liu et al., 2018).

### Exposure in Circulation

After oral administration of GQD, 174 compounds were qualitatively detected in rat plasma, urine, bile, and feces, including 107 parent and 67 biotransformed constituents (Liu et al., 2018). Moreover, the pharmacokinetics of 42 major bioactive compounds in GQD were studied (Qiao et al., 2018). The pharmacokinetic parameters of the constituents in rats that received 18.9 g/kg (1.5-fold of the clinical dosage) oral GQD are shown in Table 1. Based on the T_max_ values, after oral administration of GQD, isoquinoline alkaloids, flavonoid *O*- and *C*-glycosides, flavonoid *O*-glucuronides, and saponins sequentially reached the maximal plasma concentrations (Qiao et al., 2018). Given their dominant exposure levels (C_max_ and AUC_0-last_), puerarin and daidzein from *Puerariae Lobatae Radix*, baicalin and wogonoside from *Scutellariae Radix*, berberine and magnoflorine from *Coptidis Rhizoma*, as well as glycyrrhetinic acid and glycyrrhizic acid from *Glycyrrhizae Radix et Rhizoma Praeparata cum Melle* were the representative parent constituents that could reach systemic circulation after oral administration of GQD (Qiao et al., 2018). The elimination half-lives (T_1/2_) of most constituents of GQD were in the range of 5–10 h (Qiao et al., 2018).

## Pharmacological Effects and Mechanisms

In 2020, Prof. Heinrich et al. promoted a consensus statement on best practice in pharmacological studies (Heinrich et al., 2020). With reference to these principles, this article selected and summarized some representative pharmacological researches of GQD as follows. GQD is traditionally used to “clear internal heat” (Hai and Wei, 2009). Modern studies have shown that GQD has significant pharmacological effects on inflammatory diseases related to intestinal microbes. For example, GQD is effective against inflammatory intestinal diseases, including diarrhea (Liu et al., 2019b; Hua et al., 2019), ulcerative colitis (Xu B.-L. et al., 2015; Li et al., 2016; Fan et al., 2019; Zhao Y. et al., 2020), and intestinal adverse reactions caused by chemotherapeutic agents (Wu Y. et al., 2019). In addition, as the intestinal flora is closely related to metabolic diseases (Nicholson et al., 2012), GQD has significant effects against NAFLD (Wang Y.-l. et al., 2015; Guo et al., 2017; Guo et al., 2018; Hao et al., 2020; Zhang et al., 2020), type 2 diabetes mellitus (T2DM) (Zhang et al., 2013; Xu J. et al., 2015; Cao et al., 2020; Tu et al., 2020) and other metabolic diseases. Furthermore, based on its significant anti-inflammatory effect, GQD can be used to treat inflammatory diseases, such as lipopolysaccharide (LPS)- (Ding et al., 2020) and influenza A virus- (Shi et al., 2020) induced lung injury. Table 2 shows detailed information of the pharmacological studies carried out in model animals.

**TABLE 2:** Studies on the pharmacological effects of *Gegen- Qinlian* decoction (GQD) in model animals.

Diseases	Model animals	Treatment	Minimal active dose	Controls	Pharmacological effects	Mechanism of action	References
Diarrhea	Four piglets treated with oral *Escherichia coli*	Oral administration of 130 ml extract of 49 g GQD herbal pieces per piglet (about 2 kg) for 1 week.	130 ml extract of 49 g GQD herbal pieces per piglet (about 2 kg)	Negative control; model control	Alleviated diarrheal symptoms and intestinal mucosal injury: the histological score was reduced from higher than 6 to lower than 3 (*p* < 0.01).	Modulating gut microbiota: the relative abundance of short-chain fatty acids producing intestinal bacteria including *Akkermansia* (from 0.01 to 3.87%), *Bacteroides* (from 2.2 to 3.17%), *Clostridium* (from 0.34 to 2.39%), *Ruminococcus* (from 0.65 to 6.71%), and *Phascolarctobacterium* (from 2.09 to 4.45%) were all increased (all *p* < 0.05).	Liu et al. (2019b)
Ulcerative colitis	Ten mice treated with dextran sulfate sodium	Oral administration of GQD ethanol extract at the dosage of 0.3, 1.5, or 7.5 g/kg/day for 1 week	0.3 g/kg/day	Negative control; model control; positive control (berberine, 100 mg/kg/d)	Alleviated the severity of colitis and histopathological responses: the DAI score was reduced from about 23 to abot 13 in the minimum effective dose of GQD treated group (*p* < 0.05)	Inhibiting inflammatory responses and oxidative stress: the inflammation score was reduced from about 10 to slightly higher than 5 in the minimal active dose of GQD treated group (*p* < 0.001).	Li et al. (2016)
	Ten mice treated with dextran sulfate sodium	Oral administration of GQD ethanol extract at the dosage of 1.5, or 7.5 g/kg/day for 1 week	1.5 g/kg/day	Negative control model control; positive control (berberine, 100 mg/kg/d)	Maintained colonic mucosal homeostasis: the colon weight/colon length ratio was reduced by 1.5-fold (*p* < 0.001), while the histological score was decreased from higher than 4 to about 1 in the minimum effective dose of GQD treated group (*p* < 0.001)	Bidirectional regulation of Notch signaling: repaired the colonic mucosa through downregulation of Hes1 expression in acute UC mice, but improved the colonic mucosa through upregulation of Hes1 expression in chronic UC mice.	Zhao Y. et al. (2020)
Nonalcoholic fatty liver disease	Nine high-fat diet treated rats	Oral administration of GQD water extract at the dosage of 1.24, 3.73, or 11.2 g/kg/day for 49 days	1.24 g/kg/day	Negative control model control; positive control (pioglitazone, 5.25 mg/kg/day)	Abated liver injuries: in the minimum effective dose of GQD treated group, triglyceridewas was reduced from about 1.1 mm to about 0.8 mm (*p* < 0.05), serum total cholesterol was reduced from about 2.7 mm to about 2.1 mm (*p* < 0.05), total bile acid was reduced from about 35 μm to about 20 μm (*p* < 0.01)	Anti-oxidative stress and anti-inflammatory response involved inhibition of TLR4 signal pathways: serum levels of LPS was decreased from about 0.045 EU/ml to about 0.025 EU/ml) in the minimum effective dose of GQD treated group (*p* < 0.01).	Zhang et al. (2020)
Type 2 diabetes mellitus	Eight high-fat diet and streptozotocin-induced diabetic rats	Oral administration of 4.95, 11.55, and 18.15 g/kg for 12 weeks	4.95 g/kg/d	Negative control; model control; positive control (metformin, 200 mg/kg)	Anti-hyperglycemic and lipid lowering effects: fasting blood glucose, glycosylated serum protein, glycosylated hemoglobin, and fasting serum insulin levels were decreased (all *p* < 0.05) in the minimal active dose treated group.	Increased the protein concentration and mRNA expression of adiponectin	Zhang et al. (2013)
	Twelve high-fat diet combined with streptozotocin treated rats	Oral administration of 1.357, 4.071, and 6.785 g/kg/d for 60 days	1.357 g/kg/d	Negative control; model control; positive control (metformin, 200 mg/kg)	Anti-hyperglycemic and lipid lowering effects: fasting blood glucose was decreased from 35.75 to 25.04 mm (*p* < 0.05), while total cholesterol was decreased from about 2.5 to 1.25 mm (*p* < 0.01) in the minimal active dose treated group.	Protected pancreatic tissue and improved the insulin sensitivity index: in the minimal active dose treated group, insulin sensitivity index was decreased from higher than 2.5 to lower than 2.0 (*p* < 0.01).	Huang et al. (2017)
	Eight high-fat diet and streptozotocin induced diabetic rats	Oral administration of 11.55 ml/kg extract of 11.55 g herbal pieces of GQD twice a day for 13 weeks	11.55 ml/kg extract of 11.55 g herbal pieces of GQD twice a day	Negative control; model control; positive control (metformin, 200 mg/kg)	Anti-hyperglycemic and lipid-lowering effects: the levels FBG (from higher than 30 mm to about 20 mm), TG (from higher than 5 mm to lower than 2 mm), TC (from higher than 2.5 mm to lower than 2 mm), and LDL-C (from about 0.7 mm to about 0.5 mm) were all significantly (*p* < 0.01) decreased in the GQD treated group.	Intervening in a diverse array of PPAR-α and PPAR-γ upstream and downstream signaling transduction cascades	Tu et al. (2020)
Tumors	Four mice with xenografted RCC tumor cells	Oral administration of 150 mg/kg water extract of GQD daily for 3 weeks	150 mg/kg/d	Negative control; model control	Inhibited the expansion of renal carcinoma: tumor size was decreased from about 8 mm^3^ to about 4 mm^3^ in the minimal active dose treated group (*p* < 0.05).	Inhibited neoangiogenesis *via* decreasing the expression and activity of matrix metalloproteinase-2: number of vessels was decreased from higher than 150 to lower than 50 in the minimal active dose treated group (*p* < 0.01).	Wang N. et al. (2015)
	Twelve mice with xenografted CT26 tumor cells	Oral administration of GQD ethanol extract at the dosage of 0.3, 1.5, or 7.5 g/kg/day for 1 week	300 mg/kg/d	Negative control; model control; positive control (anti-mouse PD-1 mAb, 250 μg)	Enhanced the effect of anti-mouse PD-1 mAb on inhibiting the growth of CT26 tumors: on day 32, 0.3 g/kg/d GQD increased the tumor growth inhibition rate of PD-1 from 48.216 to 70.526% (*p* < 0.05).	Remodeling the gut microbiota and restore T-cell functions by suppressing inhibitory checkpoints	Lv et al. (2019)
Lung injury	Six mice treated with lipopolysaccharide	Pretreated with oral GQD water extract at the dosage of 500 mg/kg for 7 days	500 mg/kg/d	Negative control; model group	Reduced pulmonary edema and microvascular permeability: the protein concentration in bronchoalveolar lavage fluid was decreased from higher than 600 μg/ml to about 400 μg/ml in 500 mg/kg/d GQD treated group (*p* < 0.01).	Reducing the release of pro-inflammatory cytokines including TNF-α, IL-1β, and IL-6 (all *p* < 0.05) *via* regulating PI3K/Akt signaling pathway	Ding et al. (2020)
	Twelve influenza A virus-infected mice	Oral GQD water extract (0.19 g/d) for 5 days	0.19 g/d	Negative control; model group; positive control (oseltamivir phosphate 0.61 mg/d)	Reduced lung index from about 0.12 to 0.09 g/10 g body weight (*p* < 0.01); improved the pathological changes in the lung tissue.	Activated a balanced inflammatory response in the host to limit immune pathological injury and improve clinical and survival outcomes	Shi et al. (2020)

### Inflammatory Intestinal Diseases

Diarrhea is the eighth leading cause of death among people of all ages and the fifth leading cause of death among children younger than 5 years old (Collaborators, 2018). Diarrhea-related deaths and episodes are mainly attributed to 13 pathogens, including rotavirus and intestinal bacteria, such as *E. coli*. (Collaborators, 2018). *Via* network pharmacology analysis, 130 constituents, mainly flavonoids, alkaloids, phenyl esters, and fatty acids, were identified as the active constituents of GQD in treating rotavirus enteritis; in addition, it was supposed that GQD can effectively improve the symptoms of rotavirus enteritis by regulating calcium ions, serotonin, and gastrointestinal hormone ions, which could mutually affect the intestinal nervous system (Zhong et al., 2020). In four piglets (about 2 kg) with oral *E. coli*-induced diarrhea, compared with model control group, treatment with oral GQD (130 ml extract of 49 g GQD herbal pieces per piglet) for 1 week significantly alleviated diarrheal symptoms and intestinal mucosal injury [increased cell infiltration, Goblet cell reduction, enlargement of intercellular space, and downregulated expression of tight junction proteins (ZO-1 and occludin)] (Liu et al., 2019b). As for the histological score, it was remarkably reduced from higher than 6 in the model control group to lower than 3 in the GQD treated group (*p* < 0.01) (Liu et al., 2019b). In terms of the underlying mechanism, treatment with GQD increased the richness of intestinal bacterial species and modified the structure of gut microbial community (Liu et al., 2019b). Specifically, according to the Kruskal–Wallis test, treatment with GQD increased the relative abundance of intestinal bacteria (all *p* < 0.05), including *Akkermansia* (from 0.01 to 3.87%), *Bacteroides* (from 2.2 to 3.17%), *Clostridium* (from 0.34 to 2.39%), *Ruminococcus* (from 0.65 to 6.71%), and *Phascolarctobacterium* (from 2.09 to 4.45%), which produce short-chain fatty acids (Liu et al., 2019b). Consequently, fecal short-chain fatty acids, including acetic acid, propionic acid, and butyric acid, were increased to normal levels, further alleviating mucosal pro-inflammatory responses by inhibiting the histone deacetylase and NF-κB pathways (Liu et al., 2019b).

Ulcerative colitis, which is characterized by mucosal ulceration, rectal bleeding, diarrhea, and abdominal pain, is a chronic inflammatory bowel disease caused by multiple factors, including genetic predisposition, epithelial barrier defects, dysregulated immune responses, and environmental factors (Ungaro et al., 2017). According to a systematic review of 22 randomized controlled trials involving a total of 2028 patients with ulcerative colitis, GQD alone exhibited significant clinical effectiveness and improvement in recurrence rate (Fan et al., 2019). Furthermore, GOD has synergistic effects with modern medicines in treating ulcerative colitis (Fan et al., 2019). In a preclinical study where berberine (100 mg/kg/d) was used as positive control, oral administration of GQD ethanol extract at the dosage of 0.3, 1.5, or 7.5 g/kg/day for 1 week significantly ameliorated ulcerative colitis in ten dextran sulfate sodium-induced mice, leading to reversed body weight loss, improved state of diarrhea and prostration, ameliorated colon shortening, and reduced colonic inflammation (Li et al., 2016). To be more specific, the DAI score was remarkably reduced from about 23 in the model control group to abot 13 in the minimum effective dose (0.3 g/kg/day) of GQD treated group (*p* < 0.05) (Li et al., 2016). The protective effects of GQD were mediated *via* the inhibition of toll-like receptor (TLR) 4-mediated NF-κB signaling pathway and, consequently, the down-regulated expression of inflammatory cytokines, including tumor necrosis factor (TNF-α), interleukin (IL)-6, IL-1β, and IL-4 (Li et al., 2016). In addition, oral administration of GQD attenuated oxidative stress in the colon of ulcerative colitis model mice, as evidenced by the decrease in myeloperoxidase activity and malondialdehyde level, as well as the increase in glutathione content (Li et al., 2016). Finally, the colonic inflammation score was remarkably reduced from about 10 in the model control group to slightly higher than 5 in the minimum effective dose (0.3 g/kg/day) of GQD treated group (*p* < 0.001) (Li et al., 2016). Moreover, in a preclinical study where berberine (100 mg/kg/d) was used as positive control, oral administration of GQD ethanol extract at the dosage of 1.5, or 7.5 g/kg/day for 1 week restored the regeneration and homeostasis of the colonic mucosa by bi-directionally modulating deregulated Notch signaling in ten model mice of dextran sulfate sodium-induced acute/chronic ulcerative colitis (Zhao Y. et al., 2020). In terms of the colon weight/colon length ratio, it was reduced by 1.5- and 1.4-fold in mice in the GQD (1.5 g/kg/day) and berberine (200 mg/kg/day) groups compared with those in the chronic model control group (both *p* < 0.001). As for the histological score, it was also markedly decreased in mice in the GQD (from higher than 4 to about 1, *p* < 0.001) and berberine (from higher than 4 to about 2, *p* < 0.01) groups. GQD enhanced hypoactive Notch signaling in chronic ulcerative colitis model mice and promoted the proliferation of the epithelium and differentiation of absorption cell lines, whereas GQD suppressed hyperactive Notch signaling in acute ulcerative colitis model mice to promote the differentiation of goblet cells and secretion of protective peptides (Zhao Y. et al., 2020).

### Nonalcoholic Fatty Liver Disease

NAFLD, which is characterized by excessive fat accumulation in the hepatocytes of individuals without significant alcohol consumption, long-term use of steatogenic medication, or monogenic hereditary disorders (Chalasani et al., 2018), is a major cause of liver disease worldwide, with a global prevalence of 25.24% (Younossi et al., 2016). NAFLD can develop from simple hepatic steatosis to nonalcoholic steatohepatitis, hepatic fibrosis, hepatic cirrhosis, hepatocellular carcinoma, and liver-related mortality (Younossi et al., 2016). Oxidative stress and inflammation are two major pathological factors in the progression of NAFLD to nonalcoholic steatohepatitis (Buzzetti et al., 2016).

According to a network pharmacology research, nine compounds, including xambioona, baicalin, phaseo, worenine, wogonoside, inermine, glabrene, puerarin, and glabridin were identified as the active constituents of GQD for treating NAFLD (Hao et al., 2020). These compounds may target 17 target genes that are closely related to four pathways (AMPK, NAFLD, adipocytokine signaling, and PPAR) (Hao et al., 2020). In a preclinical study where pioglitazone hydrochloride (5.25 mg/kg/day) was used as positive control, oral administration of GQD water extract at the dosage of 1.24, 3.73, or 11.2 g/kg/day for 49 days abated liver injuries in nine NAFLD model rats by regulating lipid metabolism and reducing liver inflammation (Zhang et al., 2020). Specifically, at the minimal active dose (1.24 g/kg/day), triglyceride (TG) was reduced from about 1.1 mm to about 0.8 mm (*p* < 0.05), serum total cholesterol (CHO) was reduced from about 2.7 mm to about 2.1 mm (*p* < 0.05), total bile acid (TBA) was reduced from about 35 μm to about 20 μm (*p* < 0.01) (Zhang et al., 2020). In general, GQD treatment reduced lipid droplets in the liver; attenuated elevated serum levels of alanine aminotransferase and aspartate aminotransferase; decreased serum levels of triglyceride, cholesterol, total bile acid, low-density lipoprotein, and free fatty acid; and decreased TNF-α level in both serum and the liver (Wang Y.-l. et al., 2015; Guo et al., 2017; Guo et al., 2018; Hao et al., 2020; Zhang et al., 2020). The mechanisms of GQD were associated with an increase in the gene and protein expression of peroxisome proliferator-activated receptor gamma (PPAR-γ) (Wang Y.-l. et al., 2015), which plays a complementary role in the fine-tuning of lipid transport, catabolism, and storage (Ahmadian et al., 2013). In addition, GQD may treat NAFLD by triggering the Sirt1 pathway (Guo et al., 2017), a nicotinamide adenosine dinucleotide-dependent histone deacetylase that can inhibit inflammatory processes *via* inhibition of NF-κB transcription (Yeung et al., 2004). Given that GQD treatment decreased serum levels of LPS (reduced from about 0.045 EU/ml to about 0.025 EU/ml, *p* < 0.05), reduced inflammatory factors in LPS treated RAW264.7 cells, and inhibited free fatty acid-induced expression of TLR4 in HepG2 cells, the mechanism of action of GQD was associated with inhibition of TLR4 signaling pathways (Zhang et al., 2020). In addition, in a preclinical study where glutamine (1.5 g/kg/day) was used as positive control, oral administration of GQD water extract at the dosage of 1.26, 2.52, or 5.04 g/kg/day for 8 weeks improved HFD-induced changes in the intestinal flora of ten NAFLD model rats, leading to increased levels of *Firmicutes, Clostridia, Lactobacillus, Bacilli,* and *Erysipelotrichales*, which were similar to those of the normal control groups (Guo et al., 2018). It was known that the severity of NAFLD is closely associated with gut dysbiosis and a shift in metabolic function of the gut microbiota (Boursier et al., 2016).

### Type 2 Diabetes Mellitus

T2DM, which affects over 370 million people worldwide, is a chronic metabolic disorder characterized by the deregulation of glucose and lipid metabolism (Kahn et al., 2014). Insulin resistance or deficiency in T2DM results in the elevation of fasting and postprandial glucose and lipid levels, leading to deleterious macrovascular and microvascular outcomes as well as, eventually, a series of complications (Kahn et al., 2014).

Clinical and preclinical studies have shown that GQD is effective in treating T2DM. In a randomized, double-blinded, placebo-controlled clinical trial where 187 T2DM patients were involved, treatment with low, moderate, and high (300 ml water extract of 48, 144, and 240 g herbal pieces of GQD, respectively) dosage of oral GQD for 12 weeks improved the symptoms of patients with T2DM. In addition, GQD reduced fasting blood glucose (FBG, −1.46 ± 0.23 and −1.09 ± 0.21 vs −0.16 ± 0.22 and −0.24 ± 0.24 mm; *p* < 0.001 for high dosage vs low dosage and placebo; *p* < 0.01 for middle dosage vs low dosage and placebo) and glycated hemoglobin (HbA1c, −0.88 ± 0.14 and −0.75 ± 0.13 vs −0.35 ± 0.13 and −0.36 ± 0.15%; *p* < 0.01 for high dosage vs low dosage; *p* < 0.05 for high dosage vs placebo; *p* < 0.05, middle dosage vs low dosage and placebo) in a dose-dependent manner (Xu J. et al., 2015). In eight high-fat diet and streptozotocin-induced diabetic rats, treatment with oral GQD (4.95, 11.55, 18.15 g/kg, 200 mg/kg metformin was used as positive control) for 12 weeks improved glucose (abrogated elevations in serum FBG, HbA1c, and glycosylated protein levels, but promoted hepatic glycogen synthesis) and lipid [decreased blood triglyceride, total cholesterol (TC), and low-density lipoprotein cholesterol levels (LDL-C), but increased blood high-density lipoproteincholesterol (HDL-C) level] metabolism (Zhang et al., 2013). The minimal active dose was 4.95 g/kg, while the antidiabetic effect of 18.15 g/kg GQD was even superior to that of 0.2 g/kg metformin hydrochloride (Zhang et al., 2013). Furthermore, GQD can augment the effects of metformin (Zeng et al., 2006) and insulin (Ryuk et al., 2017).

Treatment with oral GQD water extract (23.4 g herbal pieces per killogram body weight, 300 mg/kg metformin was used as positive control) for 80 days alleviated diastolic dysfunction of the left ventricular of nine diabetic (db/db) mice by promoting myocardial glycolysis and decreasing ceramide content (Han et al., 2017). These findings suggested the efficacy of GQD in improving complications of T2DM.

Network pharmacology and bioinformatics analysis indicated that GQD regulated 82 corresponding proteins and 59 relevant biological pathways associated with diabetes (Xu et al., 2020). NMR-based metabolomic studies revealed that GQD (4 or 8 g/kg/d of oral water extract for five weeks; 300 mg/kg/d of metformin was used as positive control) significantly ameliorated disturbance in the glucose metabolism, tricarboxylic acid cycle, lipid metabolism, amino acid metabolism, gut microbial metabolism, and N-acetyl group metabolism of five to seven T2DM model rats (Tian et al., 2013).

Experiments have confirmed that GQD has the following antidiabetic mechanisms. 1) GQD improves insulin resistance. Oral administration of 1.357, 4.071, and 6.785 g/kg GQD water extract (300 mg/kg/d of metformin was used as positive control) for 60 days protected pancreatic tissue and improved the insulin sensitivity index of twelve T2DM model animals (Huang et al., 2017). For example, in the minimal active dose, i.e., 1.357 g/kg/d, treated group, insulin sensitivity index decreased from higher than 2.5 to lower than 2.0 (*p* < 0.01) (Huang et al., 2017). Integrated system pharmacology and bioinformatics analysis revealed the Hub targets (including PPARG, RELA, EGFR, CASP3, VEGFA, AR, ESR1, and CCND1) and signaling pathways (including insulin signaling, endocrine resistance, TNF signaling, PI3K-Akt signaling, AMPK signaling, MAPK signaling, NF-κB signaling, HIF-1 signaling, apoptosis, and VEGF signaling pathways) involved in the underlying mechanisms of GQD in improving diabetic insulin resistance (Cao et al., 2020). In mouse models of insulin resistance, GQD alleviated insulin resistance through silent information regulator 1 (Sirt1)-dependent deacetylation of forkhead box O1 (FOXO1) (Sui et al., 2019). In addition, PPAR-α and PPAR-γ play complementary roles in the fine-tuning of lipid transport, catabolism, and storage (Ahmadian et al., 2013). In a preclinical study where metformin (200 mg/kg) was used as positive control, oral administration of 11.55 ml/kg extract of 11.55 g herbal pieces of GQD twice a day for 13 weeks improved the symptoms of eight high-fat diet and streptozotocin induced diabetic rats by intervening in a diverse array of PPAR-α and PPAR-γ upstream and downstream signaling transduction cascades, which jointly optimized the expression of target gene profiles to promote fatty acid oxidation and accelerate glucose uptake and utilization (Tu et al., 2020). 2) GQD has anti-inflammatory and antioxidant effects. In addition to its intrinsic anti-inflammatory effects, GQD augmented the protein concentration and upregulated the expression levels of adiponectin (Zhang et al., 2013), which improves whole-body energy homeostasis; thus, GQD acts as a classic anti-inflammatory agent (Fang and Judd, 2018). 3) GQD modulates gut microbiota. In the clinical study, GQD modulated gut microbiota structure in a dose-dependent manner (300 ml water extract of 48, 144, and 240 g herbal pieces of GQD, respectively), enriching several beneficial bacteria, including *Faecalibacterium*, *Bifidobacterium*, and *Gemmiger* (Xu J. et al., 2015). In particular, the relative abundance of *Faecalibacterium prausnitzii* was negatively correlated with FBG, HbA1c, and 2-h postprandial blood glucose levels, but was positively correlated with homeostasis model assessment of β-cell function (HOMA-β) (Xu J. et al., 2015). In brief, GQD exerts antidiabetic effects through the bacteria-mucosal immunity-inflammation-diabetes axis (Gao et al., 2017).

### Tumors

GQD alone has antitumor effects. For example, although GQD showed weak cytotoxicity toward renal cell carcinoma (RCC) cells *in vitro*, compared with model group, oral administration of 150 mg/kg water extract of GQD daily for 3 weeks suppressed the growth of renal cell carcinoma (from about 8 mm^3^ to about 4 mm^3^, *p* < 0.05) in four xenograft model mice by reducing the abnormal formation of blood vessels (Wang N. et al., 2015). The effect of GQD was attributed to the inhibition of the expression and activity of matrix metalloproteinase-2 (MMP-2) (Wang N. et al., 2015), which is a critical factor for tumor neovascularization and avascular expansion (Brew and Nagase, 2010).

GQD can also be used to synergize the efficacy and reduce the adverse gastrointestinal reactions of chemotherapeutic agents. For example, therapeutic antibodies that target programmed death 1 (PD-1), an inhibitory receptor expressed by T cells, can activate therapeutic antitumor immunity (Topalian et al., 2012). Targets of GQD were related to the antitumor immune response, as shown by a systemic pharmacology analysis (Lv et al., 2019). Therefore, oral administration of GQD ethanol extract at the dosage of 0.3, 1.5, or 7.5 g/kg/day for 1 week in twelve mice bearing CT26 tumor xenograft showed synergistic effects with antibodies against PD-1 (anti-mouse-PD-1, 250 μg) by enhancing therapeutic antitumor immunity (Lv et al., 2019). For example, on day 32, 0.3 g/kg/d GQD increased the tumor growth inhibition rate of PD-1 from 48.216 to 70.526% (*p* < 0.05) (Lv et al., 2019). Combination therapy with GQD and anti-mouse PD-1 led to the downregulation of PD-1, but increased the proportion of CD8^+^ T cells in peripheral blood and tumor tissues as well as the expression of IFN-γ and IL-2 in tumor tissues, suggesting that a combination therapy could effectively restore T-cell functions by suppressing the inhibitory checkpoints (Lv et al., 2019). In addition, GQD enhanced the effect of PD-1 blockade by regulating gut microbiota and the pathways of glycerophospholipid and sphingolipid metabolism (Lv et al., 2019). In another study, life-threatening diarrhea was observed in up to 25% of patients receiving irinotecan, a widely used anticancer drug, especially for colorectal cancer (de Jong et al., 2007). Another study (0.4 mg/kg of loperamide was used as positive control) showed that oral administration of 1 g/kg and 2 g/kg of GQD water extract for five days suppressed diarrhea induced by intraperitoneal injection of 55 mg/kg irinotecan in twelve mice, mainly *via* its anti-inflammatory and antioxidant effects, which were associated with the Keap1-Nrf2 pathway (Wu Y. et al., 2019). In addition, GQD enhanced intestinal barrier function in the colon by up-regulating the expression of tight junction proteins ZO-1, HO-1, and occludin (Wu Y. et al., 2019). Moreover, GQD exerted synergistic efficacy with irinotecan in inhibiting colonic tumor growth in colorectal cancer HT-29 cell xenograft mice (Wu Y. et al., 2019). In brief, GQD not only suppressed the gut toxicity but also enhanced the antitumor effect of CPT-11, providing a useful therapeutic strategy for the clinical treatment of colon cancer (Wu Y. et al., 2019).

### Lung Injury

Acute lung injury (ALI), which is characterized by acute, progressive respiratory distress, and persistent hypoxemia, is a severe and life-threatening inflammation of the lung with high morbidity and mortality (Butt et al., 2016). Compared with model group, pretreated with oral 500 mg/kg GQD water extract for 7 days protected LPS (5 mg/kg, intraperitoneal injection) induced ALI in six BALB/c mice by reducing pulmonary edema and microvascular permeability as well as inhibiting apoptosis (Ding et al., 2020). For example, the protein concentration in bronchoalveolar lavage fluid was decreased from higher than 600 μg/ml to about 400 μg/ml in GQD treated group (*p* < 0.01). These effects were attributed to the reduction (all *p* < 0.05) in the release of LPS-induced pro-inflammatory cytokines, including TNF-α, IL-1β, and IL-6, in lung tissue, bronchoalveolar lavage fluid, and serum (Ding et al., 2020). PI3K/Akt is the main signaling pathway involved in the anti-ALI effect of GQD (Ding et al., 2020).

Seasonal influenza A virus infection, which can result in the flooding of the alveolar compartment, development of acute respiratory distress syndrome, and death from respiratory failure, is the most common cause of pneumonia-related death in the developed world (Iwasaki and Pillai, 2014). Although a robust host innate immune response helps to clear virus, it leads to severe lung injury (Iwasaki and Pillai, 2014). TLRs are the best-characterized pattern recognition receptors of the host, which initiate the immune response after influenza virus infection (Iwasaki and Pillai, 2014). In twelve influenza A virus-infected mice, on one hand, oral GQD water extract (0.19 g/d) for 5 days (0.61 mg/d oseltamivir phosphate was used as positive control) downregulated the expression of some key factors in the TLR7 signaling pathway; on the other hand, it affected the differentiation of CD4^+^ T cells (Shi et al., 2020). Hence, GQD balanced the host’s inflammatory response, which not only plays a systemic protective role in influenza virus infection but also minimizes potential immuno-pathological lung injury (Shi et al., 2020).

## Adverse Reactions

GQD showed low adverse reactions in clinical setting, except for gastrointestinal symptoms, headache, dizziness, and leukocytopenia (Tong et al., 2011; Fan et al., 2019). However, adverse reactions of the TCM compositions of GQD have been reported. For example, chronic *Glycyrrhizae Radix et Rhizoma Praeparata cum Melle* ingestion is associated with an increase in blood pressure and a decrease in plasma potassium, resulting in secondary disorders (Nazari et al., 2017). Alkaloids derived from *Coptidis Rhizoma* are potent hERG inhibitors (Schramm et al., 2011). In addition, *Coptidis Rhizoma* extract (3 g/kg) may cause acute toxicity by inhibiting acetylcholinesterase (Ma et al., 2010; Ma et al., 2011). Although this does not mean that GQD will definitely cause these adverse reactions, GQD must be used with caution before its safety is confirmed by strict evaluation.

## Discussion and Perspectives

### Effective Constituents

Network pharmacology has been used to predict the effective constituents of TCMs (Tao et al., 2013). A network pharmacology study showed that nine constituents, including xambioona, baicalin, phaseo, worenine, wogonoside, inermine, glabrene, puerarin, and glabridin, might be the effective constituents of GQD in treating NAFLD (Hao et al., 2020). In another network pharmacology analysis, quercetin, kaempferol, baicalein, wogonin, isorhamnetin, beta-sitosterol, and licochalcone A were identified as the potential bioactive compounds of GQD in treating T2DM (Cao et al., 2020). However, it should be noted that only compounds with oral bioavailability higher than 30% and drug likeness higher than 0.18 were selected for further screening in the study (Cao et al., 2020). Unfortunately, some constituents with known hypoglycemic effects, such as berberine (Lan et al., 2015) and puerarin (Zhou et al., 2014) were artificially excluded.

The material basis of TCMs should have both significant biological activity and high exposure levels at the site of action (Ma et al., 2010). In terms of regulating the intestinal flora and anti-intestinal virus, the constituents with higher exposure levels in the intestinal lumen may constitute the effective material basis of GQD. From this perspective, the constituents with relatively high content, namely puerarin, 6′-o-xylosylpuerarin, 3′-hydroxypuerarin, baicalin, 3′-methoxypuerarin, daidzin, glycyrrhizic acid, wogonoside, genistin, formonetin 8-*C*-glu (6,l)-apioside, genistein 8-*C*-apiofuranosyl (l, 6) glucoside, berberine, chrysin 6-*C*-arabinoside-8-*C*-glucoside, mirificin, and 5,7,6′-trihydroxyflavone 2′-*O*-β-d-glucopyranoside (Wang et al., 2016), could be considered as the important active constituents of GQD. Unfortunately, there are no reports on the activity of some constituents, including 6′-*O*-xylosylpuerarin, 3′-hydroxypuerarin, 3′-methoxypuerarin, formonetin 8-*C*-glu (6,l)-apioside, genistein 8-*C*-apiofuranosyl (l, 6) glucoside, chrysin 6-*C*-arabinoside-8-*C*-glucoside, mirificin, and 5,7,6′-trihydroxyflavone 2′-*O*-β-d-glucopyranoside. If intestinal barrier-protective, anti-liver disease, blood lipid-lowering, and anti-lung injury activities are considered as the pharmacodynamic indicators, the constituents that enter blood circulation and especially target tissues at high concentrations could be regarded as effective constituents. From this perspective, puerarin and daidzein from *Puerariae Lobatae Radix*, baicalin and wogonoside from *Scutellariae Radix*, berberine and magnoflorine from *Coptidis Rhizoma*, as well as glycyrrhetinic acid and glycyrrhizic acid from *Glycyrrhizae Radix et Rhizoma Praeparata cum Melle*, which both have high exposure levels and bioactivities, could be regarded as important effective constituents of GQD (Qiao et al., 2018).

In addition, the metabolites may not only be active but also have higher *in vivo* exposure levels than the parent constituents. For example, glycyrrhizic acid is almost completely metabolized to glycyrrhetinic acid during absorption (Ploeger et al., 2001), and glycyrrhetic acid is more potent than glycyrrhizic acid in some cases; for example, it is 200- to 1000-times more potent than glycyrrhizic acid in inhibiting 11-beta-hydroxysteroid dehydrogenase (Ploeger et al., 2001). Therefore, research on the *in vivo* exposure level and activity of glycyrrhetinic acid may be more important than that of glycyrrhizic acid. However, studies on the exposure levels and bioactivities of the metabolites of GQD are relatively lacking.

Identification of its effective constituents directly affects the modernization of GQD. An active constituent alignment of GQD (ACAG) was developed based on puerarin, daidzin, baicalin, berberine, palmatine, jatrorrhizine, glycyrrhizic acid, and liquiritin as the major effective constituents of GQD (Xu B. et al., 2015; Xu B.-L. et al., 2015). ACAG protected against 2,4,6-trinitrobenzene sulfonic acid-induced colitis through anti-inflammatory and antioxidant effects (Xu B.-L. et al., 2015). However, the constituents in ACAG, such as puerarin (Li et al., 2014a), daidzin (Murota et al., 2002), baicalin (Wu et al., 2014), berberine (Liu et al., 2016), and glycyrrhizic acid (Yuan et al., 2020), have poor solubility, permeability, or metabolic stability; thus, improving their oral bioavailability is challenging.

### Interactions Between the Constituents of GQD

In TCM formulas prepared *via* decoction of several herbal pieces, interactions between constituents are inevitable. First, the constituents may have synergistic or antagonistic effects at the site of action, which affects the overall efficacy. For example, in promoting glucose uptake in 3T3-L1 adipocytes and HepG2 hepatocytes, low and high doses of baicalin had additive or antagonistic effects on berberine, respectively (Zhang C.-H. et al., 2014). Furthermore, there may be synergistic or antagonistic effects between the constituents in the process of ADME, which affects the concentration of active constituents at the site of action. Many studies have been carried out in this area. For example, baicalin, berberine, and glycyrrhizin improved the permeability of puerarin across Caco-2 cell monolayers (Zhang et al., 2012). Moreover, puerarin (Zhang et al., 2012) and glycyrrhizic acid (Qiao et al., 2018) improved the permeability of berberine. Glycyrrhizic acid improved the permeability of berberine by inhibiting P-glycoprotein, a well known efflux transporter (Qiao et al., 2018).

The physical and chemical interactions between constituents are especially worthy of in-depth study. The interactions between the constituents of TCMs may change the existing form of these compounds. For example, GQD contains micro- and nano-scale aggregates that improve the bioavailability of baicalin, which is responsible for the synergistic actions between the multiple constituents (Lin et al., 2017). However, how these aggregates are formed is still unclear. Berberine could form complexes with saponins from *Glycyrrhizae Radix et Rhizoma Praeparata cum Melle* (Li et al., 2017) as well as baicalin and wogonoside from *Scutellariae Radix* (Wang et al., 2012). Berberine could self-assemble with baicalin into nanoparticles or self-assemble with wogonoside into nanofibers; both of which are mainly governed by electrostatic and hydrophobic interactions (Li T. et al., 2019). In addition, naturally occurring proteinaceous nanoparticles were identified in *Coptidis Rhizoma* extract, and they can act as carriers that facilitate berberine absorption in a concentration-dependent manner (Ma et al., 2016). *Glycyrrhizae Radix et Rhizoma Praeparata cum Melle* extract also contains large molecules, that is, *Glycyrrhizae Radix et Rhizoma Praeparata cum Melle* proteins, which have a molecular weight of approximately 31.0 kDa (Ke et al., 2015). *Glycyrrhizae Radix et Rhizoma Praeparata cum Melle* proteins can self-assemble into near-spherical nanoparticles with an average diameter of 206.2 ± 2.0 nm, which are stable at 25°C for 7 days (Ke et al., 2015). Moreover, nano-sized aggregates have been separated from *Puerariae Lobatae Radix* extract (Wang G. et al., 2015; Su et al., 2016). In fact, natural formation of nanoparticles is common in TCM extracts (Zhao Q. et al., 2020). The formation of these nanoparticles significantly changes the existing form, solubility, permeability, uptake mechanism, and transport pathway of the active constituents of TCMs, and greatly affects their pharmacokinetic and pharmacological properties (Zhao Q. et al., 2020). Taken together, these findings suggested that the physical and chemical changes that occur during the decoction of GQD, and the consequences of these changes, are worthy of in-depth study.

### Pharmacokinetics of GQD

Pharmacokinetics is an important reference for formulating a dosage regimen. However, GQD contains dozens of constituents with different pharmacokinetic parameters (Table 1). Therefore, the formulation of a GQD dosage regimen based on the T_1/2_ value of any constituent is insufficient. This is also a common problem faced in treatment with TCMs. To overcome this problem, a strategy of “integrated pharmacokinetic study of multiple constituents of TCM” was proposed (Li et al., 2008; Hao et al., 2009; Shi et al., 2018). According to this strategy, the pharmacokinetic weight coefficient of each constituent is first calculated based on AUC, a pharmacokinetic parameter that reflects the *in vivo* exposure level of a constituent, and then a mathematical model is used for integrating multiple constituents to obtain a blood concentration time curve that can characterize the overall behavior of the TCM. Finally, the integrated pharmacokinetic parameters were calculated (Li et al., 2008; Hao et al., 2009; Shi et al., 2018). The integrated pharmacokinetics of GQD in rats have been revealed using this strategy (Li et al., 2008). In view of the significant differences between the pharmacokinetic properties of flavonoids and alkaloids, the authors individually calculated the integrated pharmacokinetic parameters of flavonoids (puerarin, daidzein, baicalin, baicalein, wogonin, wogonin, liquiritin, and liquiritigenin) and alkaloids (berberine, palmatine, and jatrorrhizine) (Li et al., 2008). However, it should be noted that the T_1/2_ values of alkaloids in this study were much greater than those in other studies [25.83–29.02 h (Li et al., 2008) *vs* 1.62–11.42 h (Qiao et al., 2018) or 7.0–7.5 h (Zhang Y. et al., 2014)].

Furthermore, pharmacokinetic parameters in circulation do not fully reflect the pharmacokinetic properties of GQD. From the perspective of pharmacodynamics, GQD acts by acting on microorganisms such as bacteria and viruses in the intestinal cavity, preserving the tight junctions between intestinal wall cells, or protecting tissues, such as liver and lung tissues. Therefore, the concentration and kinetic properties of the constituents in the abovementioned targets are closely related to the efficacy of GQD. Studies have shown that some constituents of GQD have unique pharmacokinetic properties. For example, the oral bioavailability of berberine is as low as 0.36% (Liu et al., 2010), suggesting that its local action in the gut may be more important than its absorptive action. After absorption, berberine concentration in the liver is dozens of times higher than that in the blood (Liu et al., 2010), whereas its elimination in the brain is significantly slower than that in the circulation (Wang et al., 2005). In short, the dosing regimen cannot be formulated simply based on the data of pharmacokinetic parameters in blood. Therefore, pharmacokinetic studies of the effective constituents of GQD in the intestinal cavity, intestine, and target tissues would have more clinical significance.

In addition, pharmacokinetic studies in normal animals have certain limitations because pathological conditions have a significant impact on drug pharmacokinetics. For example, in minipig models of *E. coli* infection-induced diarrhea receiving oral GQD, several pharmacokinetic parameters, including AUC_0-t_, C_max_, MRT, and T_1/2_, of puerarin, wogonin, and daidzein, were different from those in normal minipigs (Ling et al., 2017). Pathological conditions often significantly affect the activity and expression of drug-metabolizing enzymes (Shah and Smith, 2015) and transporters (Evers et al., 2018), which determine the ADME and pharmacokinetics of drugs. Therefore, pharmacokinetic studies of GQD in disease models would have more clinical significance.

### Pharmacological Effects of GQD

TCMs are usually orally administered as crude extract. Due to the low contents of active constituents, the dosages of TCM extracts in model animals often reach the level of g/kg. From this point of view, the dosage of GQD in the pharmacological studies cited in this paper is in general within the acceptable range. The reported pharmacological effects of GQD were mostly produced at the same level of dosage, i.e. several g/kg. Therefore, it seems that the multiple pharmacological effects of GQD are not caused by different dosages.

Certain pharmacological effects of GQD are very significant. For example, the antidiabetic effect of 18.15 g/kg oral GQD was even superior to that of 0.2 g/kg metformin hydrochloride in diabetic rats (Zhang et al., 2013). But unfortunately, some of the studies did not design a positive control group, and other studies did not directly compare the pharmacological effects of GQD with the positive drugs.

**FIGURE 2:**
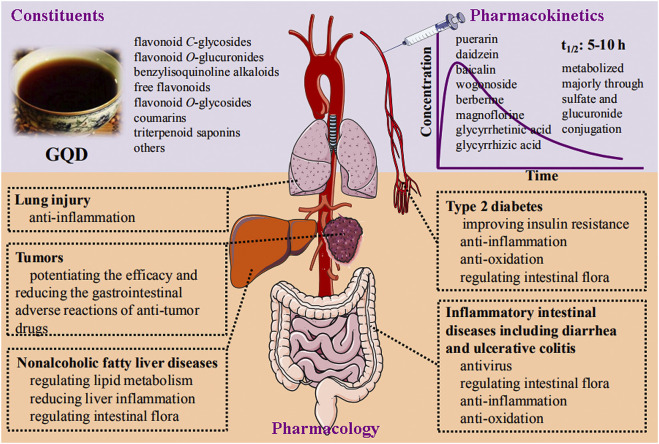
Schematic diagram of constituents, pharmacokinetics, and pharmacology of *Gegen- Qinlian* decoction.

GQD has the basic pharmacological effects of modulating gut microbiota and anti-inflammatory and antioxidant properties. These are the common mechanism of action for GQD to exert multiple pharmacological effects (Figure 2). Alterations in intestinal microbes are directly related to inflammatory intestinal diseases (Morgan et al., 2012), diabetes (Qin et al., 2012), nonalcoholic fatty liver (Mouzaki et al., 2013), lung injury (Budden et al., 2017), and tumors (Zitvogel et al., 2015). In addition, oxidative stress and inflammation are the common pathological changes of the above diseases. Therefore, it is not difficult to understand why GQD has multiple pharmacological effects.

It should be pointed out that medicinal plants are world widely used in the prevention and treatment of diseases, and are included in the Pharmacopoeias of countries such as Russia (Shikov et al., 2021), India (Prakash et al., 2017), Japan (Chen et al., 2017), Iran (Naghibi et al., 2014), and Brazil (Principe and Spira, 2009). As for the individual TCMs of GQD, *Scutellariae Radix* is officially recorded in the British Pharmacopoeia (2018) and the European Pharmacopoeia (9.0) (Wang et al., 2018), *Puerariae Lobatae Radix* is used in Japan and other East Asian countries (Wong et al., 2011). Berberine-containing botanic medicinal plants including the Indian Barberry (*Berberis aristata* DC., Berberidaceae), European Barberry (*Berberis vulgaris* L., Berberidaceae), American Hydrastis (*Hydrastis canadensis* L. or Goldenseal, Ranunculaceae) and Goldthread are widely used (Tang et al., 2009). In terms of *Glycyrrhizae Radix et Rhizoma Praeparata cum Melle*, it is one of the oldest and most popular herbal medicines in the world (Pastorino et al., 2018; Jiang et al., 2020). The individual TCMs of GQD are world widely used because of their potent pharmacological effects. For example, *Coptidis Rhizoma* has significant antibacterial (Yu et al., 2005), anti-inflammatory (Kuo et al., 2004), hypoglycemic (Yin et al., 2008), lipid-lowering (Zhang Z. et al., 2014) and anti-tumor (Tang et al., 2009) effects, *Scutellariae Radix* has significant antibacterial (Cushnie and Lamb, 2011), antioxidant (Gao et al., 1999), anti-inflammatory (Dinda et al., 2017), and anti-tumor effects (Li-Weber, 2009), *Glycyrrhizae Radix et Rhizoma Praeparata cum Melle* has antiviral and antimicrobial (Wang L. et al., 2015), and antioxidant and anti-inflammatory activities (Fu et al., 2013), and *Puerariae Lobatae Radix* is famous for its hypoglycemic effects (Wong et al., 2011). The pharmacological effects of GQD are the result of the joint actions of the four individual TCMs and their constituents. However, whether the pharmacological effects of GQD reflects the integration, addition, or synergy of these individual TCMs is worth studying. In addition, comparing the pharmacological effects of GQD and its individual TCMs would help to transfer the results of local traditional medicine to the global community.

### Potential Herb–Drug Interactions of GQD

In clinical setting, combination with GQD is usually used to enhance the efficacy and/or reduce adverse gastrointestinal reactions of modern medicines. For example, GQD is a highly effective adjunct to metformin for the treatment of T2DM (Ryuk et al., 2017). In addition, metformin can cause adverse reactions such as symptoms of gastrointestinal intolerance, including diarrhea, nausea, flatulence, indigestion, vomiting, and abdominal discomfort; GQD can alleviate these adverse effects to improve patient compliance with the therapy (Ryuk et al., 2017). Furthermore, GQD can enhance the antitumor effect of anti-mouse-PD-1 (Lv et al., 2019) and reduce gastrointestinal adverse reactions caused by irinotecan (Wu Y. et al., 2019).

However, TCMs can affect the pharmacokinetics of co-administered drugs by modulating the expression and activity of drug-metabolizing enzymes and drug transporters (Ma and Ma, 2016). Therefore, potential pharmacokinetic interactions between GQD and the combined modern medicine should be considered. GQD can inhibit five important CYP450 isoforms, that is, CYP1A2, CYP2C11, CYP2D2, CYP2E1, and CYP3A1/2 (Liu et al., 2015). In addition, GQD can inhibit the function of some uptake transporters, such as monocarboxylate transporter (MCT) (Liu et al., 2013). GQD significantly decreased the MCT-mediated uptake of valproic acid in Caco-2 cells and the oral bioavailability of valproic acid in rats (Liu et al., 2013). Furthermore, GQD downregulated the mRNA expression of efflux transporters, including P-glycoprotein and MRP1-6, in Caco-2 cells (Li et al., 2018). These findings suggested that GQD may influence the ADME (absorption, distribution, metabolism, and excretion) of co-administered drugs.

Moreover, some constituents of GQD, such as glycyrrhizic acid and glycyrrhetinic acid from *Glycyrrhizae Radix et Rhizoma Praeparata cum Melle* (Li N. et al., 2019), may modify the solubility and permeability of combined drugs. For example, glycyrrhizic acid forms supramolecular complexes with simvastatin (Vetrova et al., 2017) and increases the solubility of simvastatin by up to more than 100-fold (Kong et al., 2017). In addition, glycyrrhizic acid aggregation occurs around paclitaxel molecules in solution and can act as drug carriers for paclitaxel (Hussain, 2019). Furthermore, whether macromolecules such as polysaccharides and proteins can adsorb and change the existing form of combined drugs, similar to the protein in *Coptidis Rhizoma* extract that adsorbs berberine (Ma et al., 2016), is of great concern.

## Conclusion

In conclusion, the chemical constituents, pharmacokinetics, and pharmacological effects of GQD have been thoroughly studied (Figure 2). The main constituents of GQD could be classified into eight groups according to their structures: flavonoid *C*-glycosides, flavonoid *O*-glucuronides, benzylisoquinoline alkaloids, free flavonoids, flavonoid *O*-glycosides, coumarins, triterpenoid saponins, and others. Specifically, the constituents present at a concentration of more than 1 mg/g were puerarin, 6′-*O*-xylosylpuerarin, 3′-hydroxypuerarin, baicalin, 3′-methoxypuerarin, daidzin, glycyrrhizic acid, wogonoside, genistin, formonetin 8-*C*-glu (6,l)-apioside, genistein 8-*C*-apiofuranosyl (1,6) glucoside, berberine, chrysin 6-*C*-arabinoside-8-*C*-glucoside, mirificin, and 5,7,6′-trihydroxyflavone 2′-*O*-β-d-glucopyranoside. Most constituents of GQD have good permeability and can be absorbed throughout the intestine. The parent constituents of GQD that enter circulation mainly include puerarin and daidzein from *Puerariae Lobatae Radix*, baicalin and wogonoside from *Scutellariae Radix*, berberine and magnoflorine from *Coptidis Rhizoma*, as well as glycyrrhetinic acid and glycyrrhizic acid from *Glycyrrhizae Radix et Rhizoma Praeparata cum Melle*. Most of these constituents have a half-life of 5–10 h. The constituents are eliminated after phase I (oxidation, reduction, hydrolysis) and phase II (glucuronidation and sulfation) metabolisms. A randomized, double-blinded, placebo-controlled clinical trial verified that GQD is effective in treating T2DM. In preclinical studies, GQD is effective against diarrhea, ulcerative colitis, NAFLD, and T2DM based on its significant anti-pathogenic microorganisms (bacteria and viruses), anti-inflammatory, and antioxidant effects. GQD is also effective against LPS- and influenza A virus-induced lung injury, and can enhance the efficacy of antitumor drugs and reduce their adverse effects, especially diarrhea.

In the future, more experiments should be done on the chemical, pharmacokinetic and pharmacological interactions between the main active constituents of GQD, which would help to reveal what is happening in GQD and help to evaluate whether GQD is indeed superior to individual TCMs or even a single active constituent. It is expected that GQD would be developed from a mixture of hundreds of compounds into a natural product with simple and stable material basis and more controllable quality. In addition, although GQD has a long history of clinical medication close to 2000 years, randomized, double-blind, placebo-controlled clinical trials on GQD are still rare. There is no doubt that high-level clinical trials would greatly promote the research and development of GQD.
